# Arsenic (III) adsorption on iron acetate coated activated alumina: thermodynamic, kinetics and equilibrium approach

**DOI:** 10.1186/2052-336X-11-42

**Published:** 2013-12-20

**Authors:** Bodhaditya Das, Rashmi Rekha Devi, Iohborlang M Umlong, Kusum Borah, Saumen Banerjee, Anup Kr Talukdar

**Affiliations:** 1 Defence Research Laboratory (DRDO), Tezpur, India; 2 Department of Chemistry, Gauhati University, Guwahati, India

**Keywords:** Arsenic, Iron acetate, Activated alumina, Adsorption, Kinetic, Equilibrium

## Abstract

The adsorption potential of iron acetate coated activated alumina (IACAA) for removal of arsenic [As (III)] as arsenite by batch sorption technique is described. IACAA was characterized by XRD, FTIR, EDAX and SEM instruments. Percentage adsorption on IACAA was determined as a function of pH, contact time and adsorbent dose. The study revealed that the removal of As (III) was best achieved at pH =7.4. The initial As (III) concentration (0.45 mg/L) came down to less than 0.01 mg/L at contact time 90 min with adsorbent dose of 1 g/100 mL. The sorption was reasonably explained with Langmuir and Freundlich isotherms. The thermodynamic parameters such as *ΔG*^*0*^, *ΔH*^*0*^, *ΔS*^*0*^ and *E*_*a*_ were calculated in order to understand the nature of sorption process. The sorption process was found to be controlled by pseudo-second order and intraparticle diffusion models.

## Introduction

Arsenic contamination in natural water is the worldwide problem. There have been widespread reports of arsenic poisoning, in the major parts of Ganga delta in West Bengal [1], Brahmaputra basin [2], in northern eastern part of India, particularly Golaghat district of Assam [3] and other low-lying areas in Bangladesh [4]. The provisional standard guideline for concentration of arsenic is fixed at 10 ppb (0.01 mg/L) in the drinking water.

In natural water arsenic exists in inorganic forms with the oxidation [5] states −3, 0, +3 and +5. Arsenic is uniquely sensitive to mobilization (at pH 6.5-8.5) under both oxidizing and reducing conditions [6]. Predominantly, the species arsenite [As (III)] and arsenate [As (V)] exist in ground and surface water respectively. This is why trivalent arsenite predominates in moderately reducing anaerobic environments such as groundwater [7] and pentavalent species are stable in oxygen rich aerobic environments. It is reported that As (III) is more toxic to biological systems than As (V) [8]. Inorganic species of arsenic represents a potential threat to environment, human and animal health due to their carcinogenic and other effects. Long term drinking water exposure causes skin, lung, bladder and kidney cancer as well as pigmentation changes, skin thickening (hyperkeratosis) neurological disorders, muscular weakness, and loss of appetite [9]. It is very essential, therefore, to remove arsenic from water. Usually, a removal technique of arsenic from aqueous system should be: (i) safe in operation with respect to the maximum contaminant level, (ii) highly efficient, (iii) easy for application and (iv) low cost [10]. Conventional water treatment processes remove toxic metal ions through mechanism such as sorption and particle removal. Advanced water treatment techniques, which can be used as either a primary treatment or post treatment, involve ion exchange, reverse osmosis, adsorption, coagulation, precipitation, adsorption-co precipitation with hydrolyzing metals [11] etc. Now a day, the adsorption process is getting the best preference over other treatment processes. Available literature demonstrated that arsenic removal can be achieved by adsorption process using activated alumina [12] iron oxide-coated sand [13] iron oxide-coated cement [14] and activated red mud [15]. There is also report of arsenic removal by coagulation method using ferric chloride [16]. The removal of arsenic from drinking water using activated alumina (AA) is found to be the best removal adsorbent as per reports [17]. However, for As (III) removal, both the rate of adsorption as well as low adsorption capacity of As (III) limits the use of AA [18]. But most of the oxides of iron and manganese are available only as fine powders or are generated in aqueous suspension like hydroxides or gels [19]. Adsorbents in powder form have practical limitations, including difficulty in solid/liquid separation, low hydraulic conductivity and leaching of the metal/metal oxide along with treated water [20].

Present study was carried out to evaluate the performance of iron acetate coated activated alumina (IACAA) for As (III) removal. The process parameters such as effect of adsorbent dose, pH, initial concentration and contact time were investigated. The Langmuir and Freundlich isotherm models were tested for their applicability. Thermodynamic parameters for the process were also calculated to complete the investigation for efficacy of IACAA in removal of arsenic from contaminated water.

## Method and methodology

Arsenic trioxide, iron acetate, hydrochloric acid and sodium chloride of analytical reagent grade were procured from E. Merck (India) Ltd and used as received. Activated alumina was obtained from Loba Chemie Pvt., India with size between 70 and 230 μm. Double distilled (DD) water was used throughout for preparing solution. All the instruments used for the experimental purpose were calibrated as per recommended procedure. The initial pH of the arsenic solutions was adjusted using NaOH (0.1 M) and/or HCl (0.1 M) solutions as and when necessary and measured by Cyberscan pH 510 (Eutech) instrument. The determination of concentration of arsenic was done using AAnalyst 200 Atomic Absorption Spectrophotometer (Perkin Elmer). All the measurements were based on integrated absorbance and performed at 193.7 nm by using a quartz tube analyser (Perkin Elmer) followed by the atomization temperature 2000 K. Scanning Electron Microscope (JEOL 6390LV) was used to study the morphology of the samples. The Energy Dispersive X-rays Analysis (EDAX) attached to the SEM was used to analyze the elemental constituents of the adsorbents. Mineral phases of activated alumina, iron acetate coated activated alumina and arsenic adsorbed iron acetate coated activated alumina were characterized by powder X-ray diffraction (Bruker D8). The measurement conditions were taken as follows: anode material = Cu; K-alpha, ג = a 1.5406 Ǻ. Fourier Transform Infrared Spectrophotometer (FTIR) spectrum, (NICOLET Impact I-410) was used to scan the elements.

Preparation of IACAA was carried out in two steps. In first step, 25 g of activated alumina was impregnated with 25 mL of 1.5 M (CH_3_COO)_2_Fe in a heat resistant dish and the mixture was heated to 110°C after thorough mixing, until it became dry. In the second step, the same mixture was calcined at 400°C for 3 hours, cooled to room temperature and washed with DD water until the washed water became clear. The washed samples were dried at 110°C for 8 hours and stored in air tight containers for further use.

Batch sorption experiments were conducted to obtain rate and equilibrium data. The reaction mixture consisting 100 mL of known concentration of As (III) solution and known quantity of IACAA was shaken in a temperature controlled orbital shaker at three different temperatures of 301 K, 306 K and 311 K. Spiked water arsenic concentration was fixed at 0.45 mg/L. The effect of adsorbent dose was studied by varying the adsorbent dose from 0.1 g/100 mL to 2.5 g/100 mL and maintaining pH of the solution at 7.4 with a constant contact time of 90 min. The study of the effect of initial pH of the solutions on arsenic uptake by the adsorbent was done by using fix dose of the adsorbent at varying pH of the solutions. The effect of contact time was studied with varying contact time from 30 to 180 min keeping pH of the solutions and dose of the adsorbent constant. The sorption isotherm was also performed by mixing 1 g of IACAA with 100 mL spiked arsenic concentration at different initial concentrations of arsenic. The kinetics and thermodynamic parameters were established by conducting the experiments at different reaction times and at three different temperatures respectively.

### Desorption experiments

To determine the reusability of the IACAA samples adsorption/desorption cycles were repeated using the adsorbent sample. 100 mL solution of both NaOH and HCl (0.1, 0.3 and 0.5 M) and 1 g of the adsorbed adsorbent was used separately and agitated for about 2 hours at shaken speed of 165 rpm. The aqueous phase was then separated and concentration of arsenic in that phase was determined.

### Adsorption isotherm

#### Langmuir isotherm

The Langmuir isotherm is based on the assumption that, (i) maximum adsorption corresponds to a saturated monolayer of adsorbate molecules on the adsorbent surface, (ii) the energy of adsorption is constant and (iii) there is no transmigration of adsorbate molecules in the plane of adsorbent surface. The Langmuir isotherm [21] is expressed as:

(1) $$\frac{1}{q_{e}}=\frac{1}{q_{m}b}\frac{1}{C_{e}}+\frac{1}{q_{m}}$$

Where, *q*_*e*_ is the equilibrium quantity adsorbed (mg/g), *C*_*e*_ is the equilibrium concentration (mg/L), *q*_*m*_ is the maximum adsorption capacity (mg/g) and *b* is the Langmuir constant.

#### Freundlich isotherm

The linear form of the Freundlich isotherm [22] equation is expressed as:

(2) $$\ln q_{e}=\ln k_{f}+\frac{1}{n}\ln C_{e}$$

Where, *q*_*e*_ is adsorbed amount (mg/g), *C*_*e*_ is equilibrium arsenic concentration (mg/L), *k*_*f*_ (mg/g) is the Freundlich constant related to adsorption capacity and n is constant related to energy of intensity of adsorption.

### Dimensionless equilibrium parameter (*R*_*L*_)

In order to predict the adsorption efficiency of the isotherms process the Langmuir isotherm can be determined in terms of dimensionless equilibrium parameter or Langmuir isotherm constant parameter (*R*_*L*_) which can be related with Langmuir isotherm constant by the following equation [23].

(3) $$R_{L}=\frac{1}{1+bC_{o}}$$

Where, *b* is the Langmuir isotherm constant and *C*_*o*_ is the initial arsenic ion concentration (mg/L). The value of *R*_*L*_ indicates the shape of the isotherms. If the value 0 < *R*_*L*_ < 1 then the Langmuir isotherm is favourable, if *R*_*L*_ = 0 it is irreversible, if *R*_*L*_ = 1 it is linear and if *R*_*L*_ > 1 the isotherm is unfavourable.

### χ^2^ (Chi-square) analysis

To recognize a suitable isotherm model for the sorption process, χ^2^ analysis is to be generally carried out. The χ^2^ test statistic is basically the sum of the squares of the differences between the experimental data and the data obtained by calculating from models, with each squared difference divided by the corresponding data obtained by calculating from models. The equivalent mathematical statement is

(4) $$\;\chi^{2}=\sum \left[\frac{{\left(q_{e}-q_{e.m}\right)}^{2}}{q_{e,m}}\right]$$

Where, *q*_*e,m*_ is equilibrium capacity obtained by calculating from the model (mg/g) and *q*_*e*_ is the experimental data of the equilibrium capacity (mg/g). If the data from the models are similar to the experimental data, *χ*^2^ will be a small number, but if they differ then *χ*^2^ will be a bigger number [24].

### Thermodynamic investigations

Thermodynamic parameters associated with adsorption viz. standard free energy change (*ΔG*^*o*^), standard enthalpy change (*ΔH*^*o*^), standard entropy change (*ΔS*^*o*^), activation energy (*E*_*a*_), were calculated as follows:

The free energy of sorption process, considering the sorption distribution coefficient *K*_o_, is given by the equation:

(5) $$\Delta G^{0}=-\mathrm{RTln}K_{0}$$

Where, *ΔG*^*o*^ is the standard free energy change (kJ/mol), *T* is the temperature in Kelvin and *R* is the universal gas constant (8.314 J/mol/ K). The sorption distribution coefficient *K*_o_ for sorption reaction was determined from the slope of the plot $\ln\left(\frac{q_{e}}{C_{e}}\right)$ against *C*_*e*_ at different temperatures and extrapolating to zero *C*_*e*_ according to the method suggested by Khan and Singh [25].

The sorption distribution coefficient may be expressed in terms of *ΔH*^*o*^ and *ΔS*^*o*^ as a function of temperature.

(6) $$\ln K_{0}=\frac{\Delta H^{0}}{\mathrm{RT}}+\frac{\Delta S^{0}}{R}$$

Where, *∆H*^0^ is the standard enthalpy change (kJ/mol) and *∆S*^0^ is the standard entropy change (kJ/mol/K). The values of *∆H*^0^ and *∆S*^0^ can be obtained from the slope and intercept of a plot of 1n *K*_0_ against $\frac{1}{T}\;$ respectively.

A modified Arrhenius-type equation related to the surface coverage (*θ*) is expression for sticking probability, S^*^. This is a function of the adsorbate/adsorbent system, which is the measure of the potential of an adsorbate to remain on the adsorbent indefinitely [26] and it can be expressed as:

(7) $$S^{*}=\left(1-\theta \right)\exp-\left(\frac{E_{a}}{\mathrm{RT}}\right)$$

Where, *θ* is surface coverage,

(8) $$\theta =\left(1-\frac{C_{e}}{C_{0}}\right)$$

Where, *C*_0_ and *C*_*e*_ are the initial and equilibrium arsenic concentrations respectively. The plot of ln(1–*θ*) against $\frac{1}{T}$ will give a linear plot with intercept of lnS* and slope of $\frac{E_{a}}{R}$.

### Adsorption kinetics

In order to investigate the sorption mechanism for removal process, pseudo first and second-order kinetic models are used at different experimental conditions. A simple pseudo-first-order kinetic model [27], is represented as:

(9) $$\log\left(q_{e}-q_{t}\right)=\log q_{e}-\frac{K_{\mathrm{ad}}}{2.303}t$$

Where *q*_*t*_ is the amount of adsorbate on the surface of the adsorbent at time *t* (mg/g) and *K*_*ad*_ is the equilibrium rate constant of pseudo-first-order sorption (min^-1^).

In addition, pseudo-second-order model is also widely used. Though there are four types of linear pseudo-second-order kinetic models [28], the most popular linear form used has the equation:

(10) $$\frac{t}{q_{t}}=\frac{1}{h}+\frac{t}{q_{e}}$$

Where, $q_{t}=q_{e\;}^{2}\mathrm{kt}/\left(1+q_{t}\mathrm{kt}\right)$, the amount of adsorbate on the surface of the adsorbent at any time, *t* amount (mg/g), *k* being pseudo-second-order rate constant (g/mg min), *q*_*e*_ is the amount of adsorbate sorbed at equilibrium (mg/g)and the initial sorption rate, *h* = $kq_{e}^{2}$(mg/g min). The value of *q*_*e*_ (1/slope), *k* (slope^2^/intercept) and *h* (1/intercept) of the pseudo-second-order equation were obtained experimentally by plotting $\;\frac{t}{q_{t}}$ against *t* for arsenic sorption at different temperatures.

### Intraparticle diffusion model

The contact time experimental results can be used to study the rate-limiting step in the adsorption process, [29] and its equation can be expressed as:

(11) $$\;q_{t}=k_{i\;}t^{1/2}$$

Where, *k*_*i*_ is the intraparticle rate constant (mg/g min ^0.5^). The slope of plot of *q*_*t*_ 0versus *t*^1/2^ gives the value of the intraparticle rate constant.

## Results and discussion

### Effect of adsorbent dose

**Figure 1:**
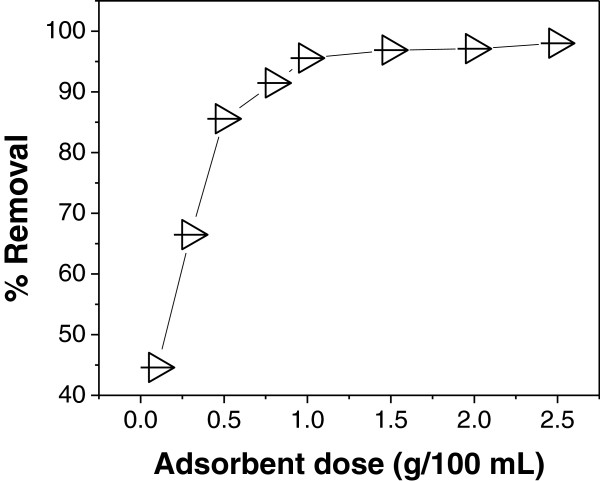
Effect of adsorbent dose on As (III) adsorption at As(III) concentration = 0.45 mg/L, pH = 7.4 and contact time = 90 min.

The effect of adsorbent dose on arsenic removal at fixed initial arsenic concentration is shown in Figure 1. It was observed that the adsorption efficiency increases rapidly with increase in adsorbent dose from 0.1 – 1 g/100 mL; a marginal increase is observed on further increase in the adsorbent dose. The removal efficiency was observed to increase sharply up to 1 g/100 mL of As (III) solution thereafter removal becomes almost constant. Thus, 1 g/100 mL was taken for other studies. The increase in the efficiency of removal may be attributed to the fact that with the increase in adsorbent dose, more adsorbent surface is available for the solute to be adsorbed [30].

### Effect of pH

**Figure 2:**
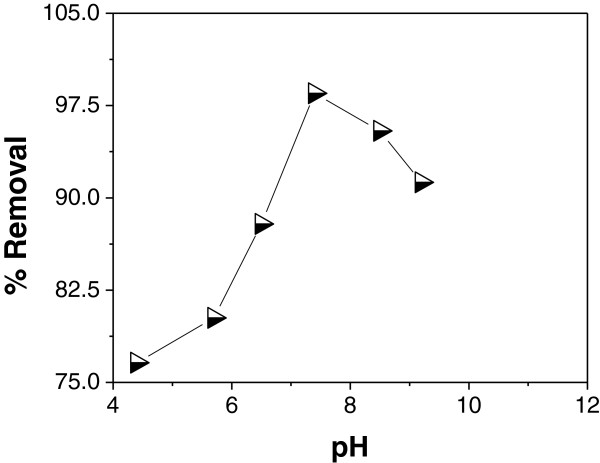
Effect of pH on As (III) adsorption at As(III) concentration = 0.45 mg/L, adsorbent dose = 1 g/100 ml and contact time = 90 min.

The effect of pH on the adsorption of As (III) onto IACAA was studied in the pH range of 4.2 to 9.2. Figure 2 reveals that the efficiency of As (III) removal increases with increasing pH from 4.2 to 7.4. In the present study best arsenic removal of 98.5% has been achieved at pH = 7.4. The removal efficiency of IACAA is found to decrease both at lower and higher pH from 7.4. The highly protonated surface of IACAA is not favourable for As (III) removal resulting almost no change in the extent of adsorption within the pH range 4.2 to 6.1. With increase in pH of the system, the degree of protonation of the surface reduces gradually and approaches to zero near zero point charge (ZPC) of the adsorbent. The species like H_2_AsO_4_ ^-^ and HAsO_4_ ^2-^ which may be formed during the adsorption process may play a role in the molecule surface interaction or occlusion phenomena and may also affect the arsenic adsorption mechanism [31].

### Effect of contact time

**Figure 3:**
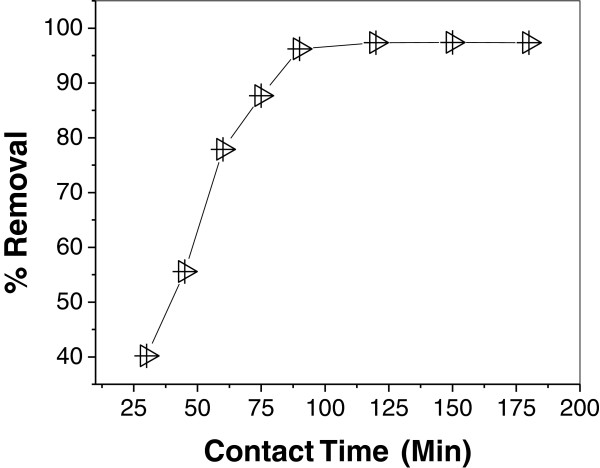
Effect of contact time on As (III) adsorption at As(III) concentration = 0.45 mg/L, adsorbent dose = 1 g/100 L and pH = 7.4.

Figure 3 demonstrates the effect of contact time on the adsorption of As (III) onto IACAA. From the figure it is clear that adsorption efficiency increases with increasing contact time, reaching a maximum removal at contact time of 90 minutes; thereafter removal becomes linearly constant. This may be due to the overlapping of active sites with arsenic species as the contact time increases and also due to decrease in the effective surface area resulting in the conglomeration of exchange particle [32].

### Effect of initial concentration

**Figure 4:**
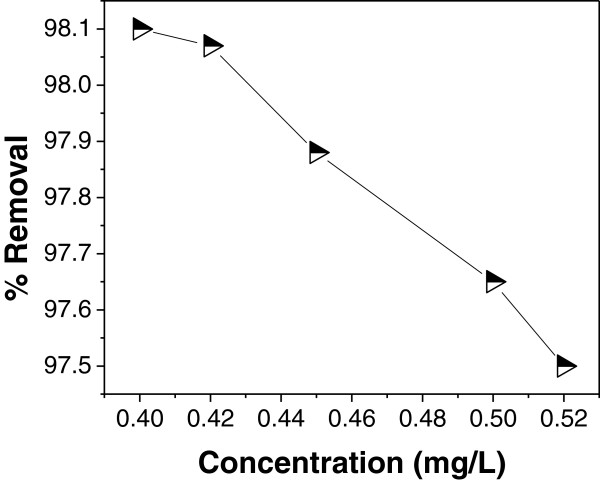
Effect of initial concentration on As (III) adsorption at pH = 7.4, adsorbent dose = 1 g/100 ml and contact time = 90 min.

Effect of initial concentration on the percentage removal of As (III) was studied by varying different initial arsenic concentration. The effect of initial concentration on removal of As (III) was shown in Figure 4 which reveals that removal efficiency is higher with lower initial arsenic concentration (0.40 mg/L); a gradual decrease in As (III) uptake by IACAA was observed with increasing feed concentration of As (III). The reason for the decrease in As (III) adsorption efficiency at higher initial concentration may be due to fact that the active sites ultimately becomes saturated with adsorbed arsenic and further addition of arsenic to the solution would not be expected to increase the amount to be adsorbed significantly [33].

### Analysis studies

**Figure 5:**
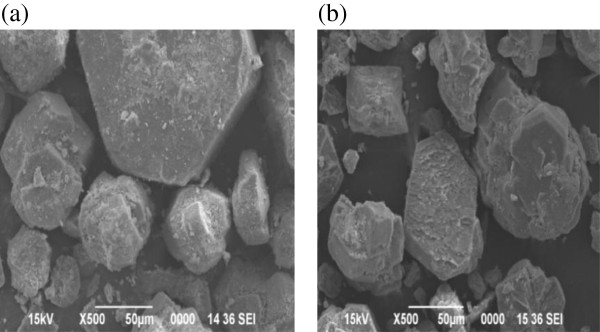
**SEM image of IACAA.** SEM of coated activated **(a)** before sorption of arsenic &**(b)** after sorption of arsenic.

**Figure 6:**
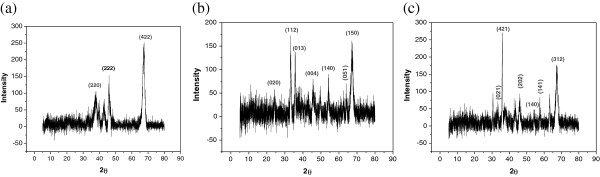
X-Ray spectra of Activated Alumina and IACAA. **(a)** Activated Alumina **(b)** IACAA before As (III) adsorption **(c)** IACAA after As (III) adsorption.

**Figure 7:**
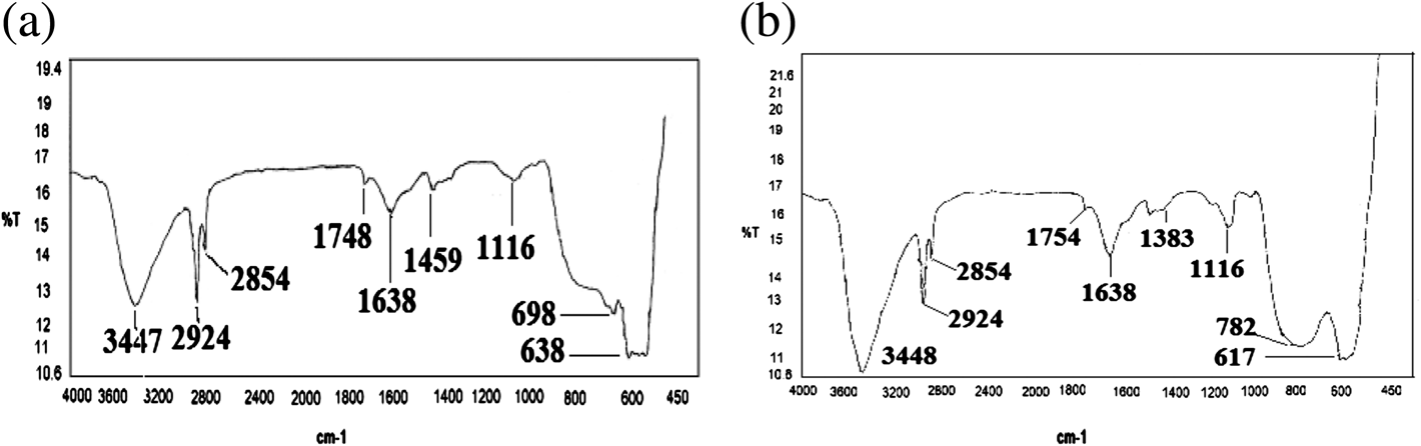
FTIR spectra of IACAA. **(a)** IACAA before As (III) adsorption **(b)** IACAA after As (III) adsorption.

**Figure 8:**
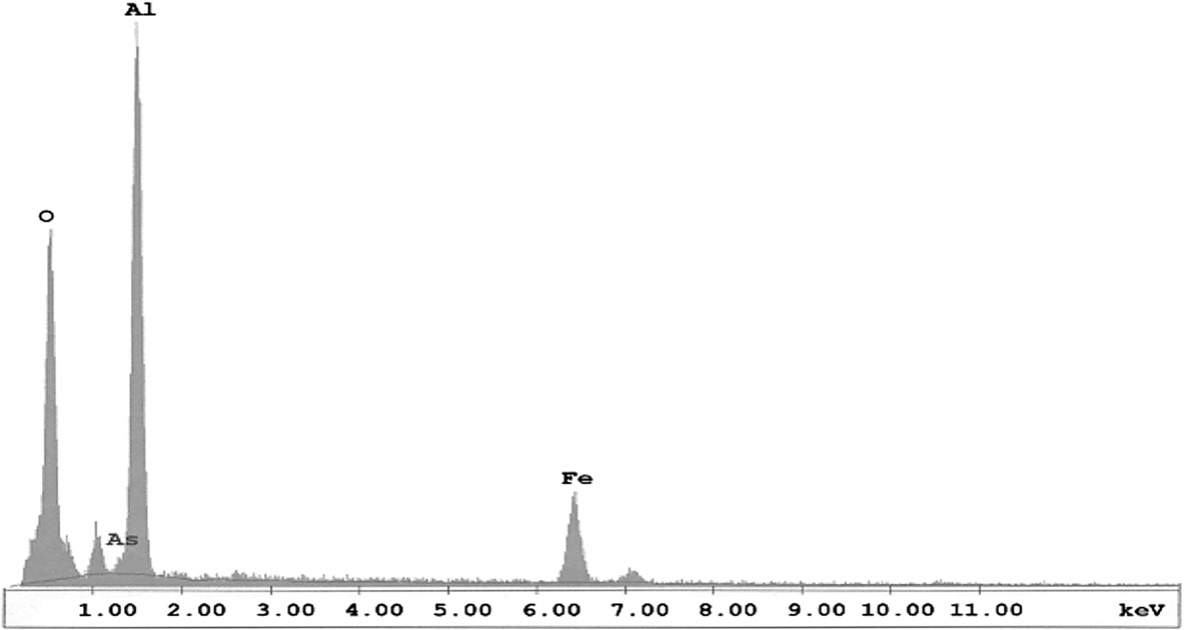
% Elemental of IACAA adsorbents after adsorption of arsenic analyzed by EDAX.

The surface morphology of iron acetate coated activated alumina (IACAA) adsorbent before and after treatment with arsenic samples were examined by scanning electron microscope (SEM). SEM photographs before and after treatment with arsenic are given in Figures 5(a) and (b) respectively. It is clear that the surface morphology of these two samples is different which confirms the arsenic sorption onto the IACAA adsorbents. X-ray diffraction patterns of activated alumina, IACAA and arsenic adsorbed IACAA are given in Figures 6(a), (b) and (c) respectively. Crystalline phases were identified by software database published by the Joint Committee on Powder Diffraction Standards (JCPDS). The main mineral phases of alumina, iron and arsenic sorption on IACAA respectively were identified as Al_2_O_3_ (JCPDS-79-1557), AlFeO_3_ (JCPDS-84-2154) and FeAsO_4_ (JCPDS-78-1545). The FTIR spectra of IACAA before and after adsorbing As (III) in aqueous solution are shown in Figures 7(a) and (b). In the Figure 7(a) IR spectrum of IACAA shows distinct bands at 1748, 1638, 1459 and 1383 cm^-1^. As described elsewhere [34], these bands arise from Fe-OH stretching and banding vibration from part of hydroxyl groups, which are converted from the iron oxide in the forms of transient complex species such as Fe-OH, Fe(OH)_2_ or FeO(OH) on the surface of IACAA. The As (III) adsorption leads to promote intensity of the spectrum by an order of magnitude without many changes in the individual positions of bands. But the IR band in Figure 7(a) that shifts from 698 cm^-1^ in case of IACAA to 782 cm^-1^ upon As (III) adsorption is attributed to the As-O stretching band following partial substitution of Fe^3+^ by As^3+^. These results are in reasonable agreement with the earlier studies of As (III) sorption to Fe and Al oxide [35]. EDAX analysis of adsorbents after adsorption of arsenic showed the presence of oxygen, arsenic, alumina and iron. The graphical representation is depicted in Figure 8.

### Adsorption studies

**Figure 9:**
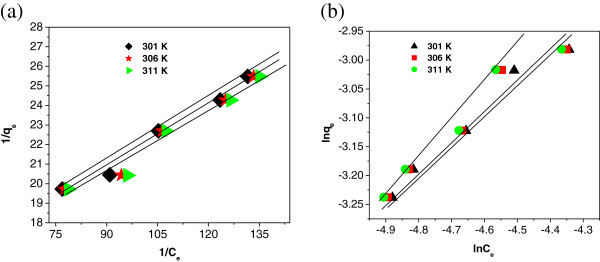
**Adsorption isotherm models of IACAA. (a)**: Langmuir isotherm of IACAA. **(b)**: Freundlich isotherm of IACAA.

The linear plot of 1/*C*_*e*_ versus 1/*q*_*e*_ of Langmuir isotherm Figure 9(a) with higher R^2^ value indicates the monolayer adsorption on IACAA. The values of *q*_*m*_ and *b* were calculated from the slope and intercept respectively are presented in Table 1.

**Table 1:** A comparison of Langmuir and Freundlich isotherm parameters obtained at different temperatures

**Temp (K)**	**Langmuir isotherm parameters**				**Freundlich isotherm parameters**				
	***q***_***m***_**(mg/g)**	***b*** **(L/mg)**	**R**^**2**^	**SD**	**1/*n***	***n***	***k***_***f***_**(mg**^**1-1/n**^**L**^**1/n**^**g**^**-1**^**)**	**R**^**2**^	**SD**
301	0.0897	0.1035	0.9918	0.3776	0.4899	2.04	0.8374	0.9877	0.0212
306	0.0903	0.1038	0.9844	0.5053	0.4888	2.05	0.8332	0.9776	0.0274
311	0.0909	0.1041	0.9823	0.5336	0.4928	2.03	0.8128	0.9754	0.0288

Temp: Temperature.

In case of Freundlich isotherm, the value of *k*_*f*_ and *n* (Table 1) are obtained from the linear plot of *ln q*_*e*_ vs *ln C*_*e*_ Figure 9(b). Value of *n* lying between 1 and 10 also indicates the favourable conditions for adsorption isotherms as well.

Langmuir isotherm constant parameter, *R*_*L*_ values at different temperatures studied were calculated and are given in Table 2.

**Table 2:** **The** ***R***_***L***_**and Chi-square values of IACAA**

**Temp**	***R***_***L***_**values**					**χ**^**2**^**values for isotherms**	
**(K)**	**0.40 mg/L**	**0.42 mg/L**	**0.45 mg/L**	**0.50 mg/L**	**0.52 mg/L**	**Langmuir**	**Freundlich**
301 K	0.0236	0.0225	0.0210	0.0189	0.0182	5.01E-05	7.93E-01
306 K	0.0235	0.0224	0.0209	0.0188	0.0181	8.04E-05/	7.84E-01
311 K	0.0234	0.0223	0.0208	0.0187	0.0180	9.02E-05	7.32E-01

R^2^ values for Langmuir isotherm (0.991, 0.984 and 0.982) at different temperatures 301 K, 306 K and 311 K are presented in Table 1. It was found out that Langmuir adsorption model is better fitted than Freundlich model. The *χ*^2^ values calculated using equation (4) is given in Table 2. In case of Langmuir isotherm, the *χ*^2^ values are found to be much lower than that of Freundlich isotherm and hence the adsorption of arsenic on IACAA follows preferably Langmuir isotherm.

### Thermodynamic studies

The thermodynamic parameter viz. ∆*G*^0^, ∆*H*^0^, ∆*S*^0^ and *E*_*a*_ were calculated with the help of equations (5, 6, 7, 8) and are presented in Table 3. The negative value of standard free energy and positive value of entropy change indicate the spontaneity of sorption of arsenic. The value of enthalpy change was positive, indicating that the sorption process is endothermic [36]. The positive value of ∆*S*^0^ shows the increasing randomness during the sorption of arsenic on IACAA. The value of S^*^ is found to be 0.011 which is very close to zero indicating that adsorption mechanism follows chemisorptions [37].

**Table 3:** Thermodynamic parameters obtained at three different temperatures during arsenic sorption on IACAA

	**Thermodynamic parameters**				
**Temp. (K)**	***ΔG***^***o***^**(KJ/mol)**	***ΔH***^***o***^**(kJ/mol)**	***ΔS***^***o***^**(kJ /mol/K)**	***Ea*** **(kJ/mol)**	S*
301	−9.84	2.0	0.0390	1.66	0.0110
306	−10.04				
311	−10.23				

### Kinetics studies

**Figure 10:**
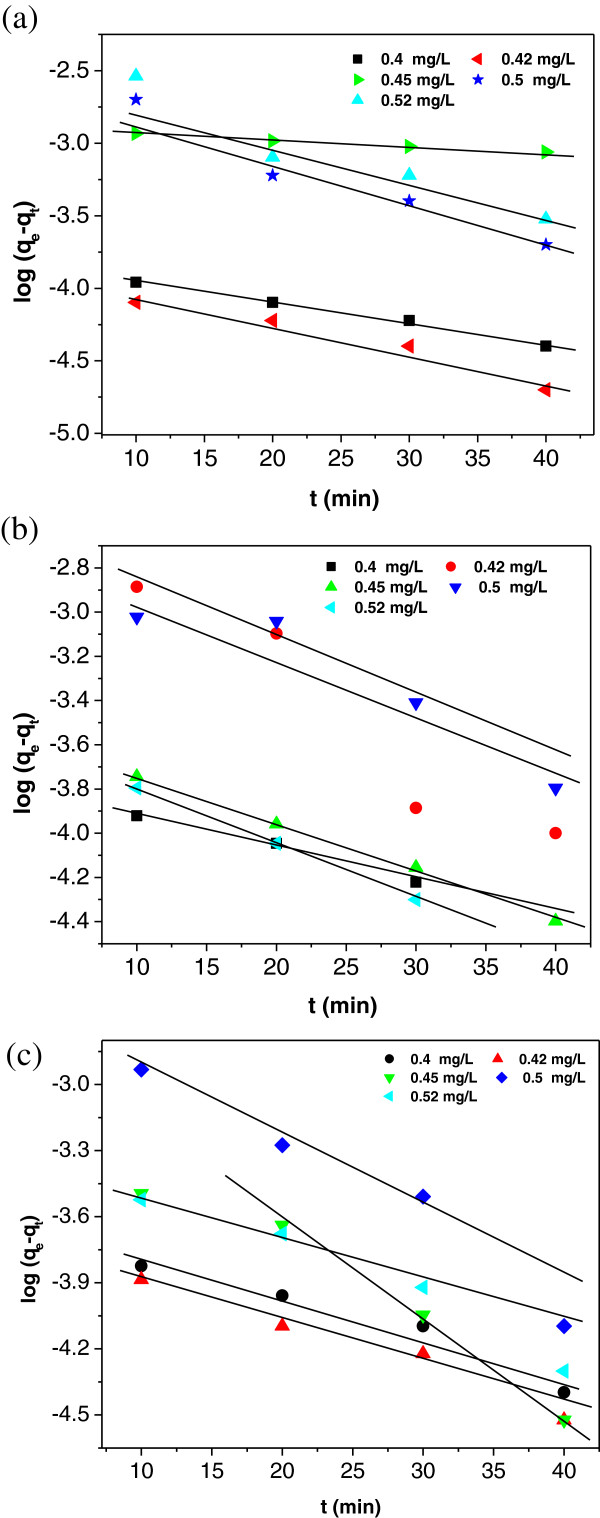
**Lagergren plot of IACAA at three different temperatures. (a)**: Lagergren plot of IACAA at 301 K. **(b)**: Lagergren plot of IACAA at 306 K. **(c)**: Lagergren plot of IACAA at 311K.

The Lagergren plots of log(*q*_*e*_–*q*_*t*_) versus *t* obtained from the equation (9) for various temperatures viz. 301, 306 and 311 K are given in Figures 10(a), (b) and (c). The values of *K*_*ad*_ at three different temperatures were calculated from slopes of the respective linear plots and also the correlation coefficient (R^2^) were computed and the values are given in Table 4.

**Table 4:** Lagergren constants for sorption of arsenic on IACAA at different temperature

***C***_***0***_	**Mass**	**301 K**		**306 K**		**311 K**	
**(mg/L)**	**(g)**	***K***_***ad***_	**R**^**2**^	***K***_***ad***_	**R**^**2**^	***K***_***ad***_	**R**^**2**^
		**(min**^**-1**^**)**		**(min**^**-1**^**)**		**(min**^**-1**^**)**	
0.40	1.0	0.0333	0.9914	0.0583	0.8531	0.0434	0.9355
0.42	1.0	0.0464	0.9411	0.0952	0.8703	0.0473	0.9612
0.45	1.0	0.0672	0.9755	0.0495	0.9972	0.0811	0.9311
0.50	1.0	0.0733	0.9330	0.0622	0.8530	0.0862	0.9463
0.52	1.0	0.0712	0.8963	0.0686	0.9784	0.0594	0.9444

The plot of *t* vs. $\frac{t}{q_{t}}$ from equation (10) gives a straight line with higher correlation coefficient r values, which is higher than that observed pseudo-first-order model indicating the applicability of the pseudo-second-order model. These values are shown in Table 5. The value of *q*_*e*_ was found to increase with increasing initial As (III) concentration and temperature. The values of rate constant (*k*) have also been found to increase with increasing temperature thus indicating chemisorption.

**Table 5:** Pseudo-second-order kinetic parameters of IACAA

**Temp**	**Conc**	***q***_***e***_	***k***	***h***	**R**^**2**^
**(K)**	**(ml/L)**	**(mg/g)**	**(g/mg min)**	**(mg/g min)**	
301	0.40	0.0382	0.5530	0.8520	1
	0.42	0.0422	0.6640	1.1280	1
	0.45	0.0451	0.7010	0.9310	0.999
	0.50	0.0492	0.8280	0.6220	0.998
	0.52	0.0451	0.9230	0.5410	0.997
306	0.40	0.0392	0.9531	1.3951	1
	0.42	0.0411	0.9770	1.6941	1
	0.45	0.0426	0.9862	0.9340	0.999
	0.50	0.0432	1.2100	0.7821	0.981
	0.52	0.0512	1.2240	0.7540	0.999
311	0.40	0.0399	0.9870	1.4370	0.999
	0.42	0.0413	0.9883	1.5031	0.998
	0.45	0.0416	1.3010	1.6010	1
	0.50	0.0419	1.3210	1.4130	0.999
	0.52	0.0507	1.3301	1.6110	1

**Figure 11:**
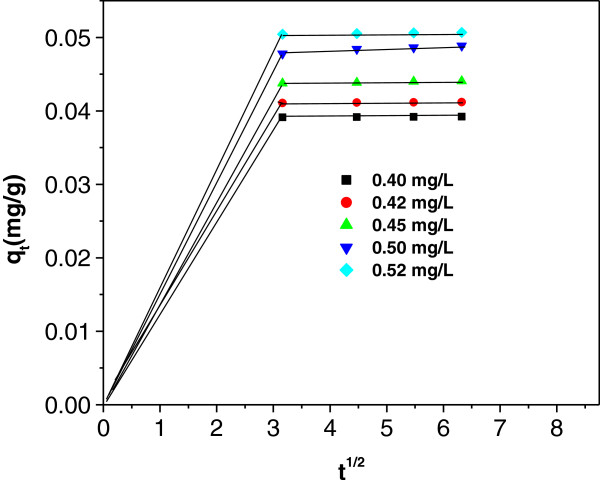
Validation of Weber-Morris equation for arsenic sorption on IACAA.

The plot of *q*_*t*_ versus *t*^1/2^ from equation (11) is shown in Figure 11. All the plots have the same general features of initial curved portion followed by linear portion and a plateau. The initial portion is attributed to the bulk diffusion and subsequent linear portion is attributed to the intraparticle diffusion. The fitness of particle diffusion model gives further evidence that the arsenic removal is a surface process under the studied experimental conditions.

The percentage removal of IACAA on As (III) was found to be higher as compared with percentage removal using Iron Oxide Coated Sand [30], Activated Alumina [38] and Iron oxide Impregnated Activated Alumina [39] as adsorbents and the list is given in Table 6.

**Table 6:** Comparison of removal efficiency of As (III) using different adsorbents

**Name of adsorbent**	**% removal**	**Experimental condition**	**References**
AA^a^	94.4	pH: 6.0-8.0, Temp^*^: 298 K	[38]
IOIAA^b^	96.7	pH: 12, Temp: 298 K	[39]
IACAA^c^	98	pH: 7.4, Temp: 301 K	Present study
IOCS^d^	88	pH: 7.5, Temp: 300 ± 2 K	[30]

^a^Activated Alumina, ^b^Iron oxide Impregnated Activated Alumina, ^c^Iron Acetate Coated activated Alumina & ^d^Iron Oxide Coated Sand; *Temperature.

### Desorption study

It was observed that no desorption of As (III) was detected under normal condition. The experimental results revealed that eluent NaOH are found to be more effective to desorp arsenic in comparison to that of HCl. The trend of desorption percentage of different concentration of NaOH is as follows: 0.1 M < 0.3 M < 0.5 M. The maximum desorption of arsenic was found to be 34.4% with 0.5 M NaOH solution.

## Conclusion

The overall study reveals that the adsorption of arsenic onto IACAA is found to be dependent on pH, adsorbent dose and contact time. Best removal of As (III) is achieved at pH = 7.4. The initial As (III) concentration (0.45 mg/L) comes down to less than 0.01 mg/L with the minimal adsorbent dose (1 g/100 mL) at contact time 90 minutes. The thermodynamic studies of sorption of arsenic on IACAA show that the reaction is spontaneous and endothermic process. The equilibrium data are fitted to both Langmuir and Freundlich adsorption isotherm. But it is found that Langmuir isotherm model fitted well followed by Freundlich. The pseudo-second order kinetic model is found to be the best correlation of the data for sorption of arsenic on IACAA. The kinetic of the reaction follows intraparticle diffusion model.

## Competing interests

The authors declare that they have no competing interests.

## Authors’ contributions

BD was the main investigator. He participated in the study design, data analysis and drafting of manuscripts. RRD was involved in experimental studies including interpretation of the results of FTIR and XRD. IMU carried out detailed adsorption, thermodynamic and kinetic studies and their interpretation. KB had done the quantitative analysis of arsenic and also extended help in other laboratory studies related to the manuscript. SB was the main advisor, helped in study design and supervised the study. AKT was responsible for data processing, support in interpretation of results and proof reading of the manuscript. All authors read and approved the final manuscript.
